# Apoptotic Gene Expression in HepG2 Cells Treated with *Ornithogalum sigmoideum* and *Smilax excelsa* Compounds

**DOI:** 10.3390/ijms27146435

**Published:** 2026-07-20

**Authors:** Onur Dirican

**Affiliations:** 1 Department of Pathology Laboratory Techniques, Vocational School of Health Services, Istanbul Gelişim University, 34310 Istanbul, Türkiye; odirican@gelisim.edu.tr; 2 Life Sciences and Biomedical Engineering Application and Research Centre, Istanbul Gelisim University, 34310 Istanbul, Türkiye

**Keywords:** medicinal plants, HepG2 cell line, cytotoxicity, mRNA expression, apoptosis

## Abstract

The present study investigates the pro- and anti-apoptotic responses of HepG2 liver cancer cells to *Ornithogalum sigmoideum* (*O. sigmoideum*) and *Smilax excelsa* (*S. excelsa*) extracts, aiming to identify their potential as anti-cancer agents. Methanolic extracts of *O. sigmoideum* and *S. excelsa* were prepared, and their phytochemical profiles were analyzed by Gas Chromatography–Mass Spectrometry (GC-MS). Cytotoxicity and IC50 values were determined in HepG2 cells using the MTT assay. The relative mRNA expression of apoptotic genes (BAX, BCL-2, and Caspase-3) was quantified by qPCR, and the treatment effect size was calculated using Cohen’s d. GC-MS analysis revealed distinct phytochemical profiles; *S. excelsa* was rich in phenolic compounds, while fatty acid esters and alcohols dominated *Ornithogalum* extracts. *O. sigmoideum* bulb extract exhibited the strongest cytotoxicity (IC50 = 125.57 µg/mL), followed by the leaves (IC50 = 156.43 µg/mL), whereas *Smilax* showed minimal toxicity (IC50 = 304.15 µg/mL). Mechanistically, the *O. sigmoideum* leaf extract showed gene expression patterns consistent with pro-apoptotic signaling, including upregulation of BAX and Caspase-3 mRNA. *O. sigmoideum* extracts, especially from leaf parts, exhibit significant cytotoxicity, and the transcriptomic profile is consistent with apoptotic pathway activation in HepG2 cells, positioning it as a candidate for further mechanistic investigation.

## 1. Introduction

Liver cancer remains a major global health challenge and is currently the sixth most commonly diagnosed malignancy and the fourth leading cause of cancer-related mortality worldwide. Among primary liver cancers, hepatocellular carcinoma (HCC) accounts for approximately 80–90% of cases and represents the most prevalent histological subtype. HCC is characterized by aggressive tumor progression, high recurrence rates, and poor prognosis, largely due to late-stage diagnosis and limited treatment responsiveness [1].

Current therapeutic strategies for HCC include surgical resection, liver transplantation, locoregional therapies, and systemic treatments such as targeted therapies and immunotherapeutic agents. Despite these advances, treatment outcomes remain unsatisfactory for many patients. High recurrence rates, drug resistance, and significant systemic toxicity limit the long-term effectiveness of existing therapeutic approaches [2]. Consequently, there is a growing need to identify novel therapeutic strategies that are both effective and safer, particularly those capable of targeting multiple molecular pathways involved in tumor progression [3,4,5].

One of the hallmarks of cancer development is the disruption of apoptosis, a tightly regulated form of programmed cell death responsible for maintaining cellular homeostasis. In hepatocellular carcinoma, dysregulation of apoptotic pathways allows malignant cells to evade death signals and contributes to uncontrolled proliferation and therapeutic resistance. Key regulators of the intrinsic apoptotic pathway include members of the Bcl-2 protein family and caspase enzymes. Anti-apoptotic proteins such as Bcl-2 promote cell survival, whereas pro-apoptotic proteins such as BAX and executioner enzymes, including Caspase-3, facilitate programmed cell death [6]. The imbalance between these pro- and anti-apoptotic factors plays a critical role in hepatocarcinogenesis. Therefore, restoring the apoptotic balance has become an important strategy in the development of new anticancer therapies.

In recent years, natural products and plant-derived compounds have gained increasing attention as potential sources of novel anticancer agents. Their structural diversity, ability to interact with multiple molecular targets, and generally lower toxicity profiles make them attractive candidates for drug discovery [7,8]. Numerous phytochemicals have been reported to induce apoptosis in cancer cells through mechanisms involving mitochondrial dysfunction, activation of caspase cascades, and modulation of Bcl-2 family proteins [9,10]. As a result, medicinal plants continue to represent an important reservoir of bioactive molecules for the development of innovative therapeutic agents.

Within this context, species belonging to the genera *Ornithogalum* and *Smilax* have attracted considerable scientific interest due to their pharmacologically active phytochemical content. Several *Ornithogalum* species have been reported to contain steroidal glycosides, flavonoids, and other bioactive compounds that exhibit cytotoxic, antioxidant, and pro-apoptotic activities in various cancer cell models [11,12,13,14]. Similarly, species of the genus *Smilax* are rich in flavonoids, phenolic compounds, and saponins and have demonstrated antioxidant, anti-inflammatory, antimicrobial, and anticancer properties in different experimental systems [15,16,17,18]. Despite these promising findings, the biological activities of specific species, such as *Ornithogalum sigmoideum Freyn & Sint* (*O. sigmoideum*) and *Smilax excelsa* L. (*S. excelsa*), remain insufficiently characterized, particularly regarding their potential role in modulating apoptotic pathways in HCC cells.

Importantly, the molecular mechanisms through which these plant extracts influence apoptosis-related gene expression in liver cancer models have not yet been systematically investigated. This represents a significant knowledge gap, especially considering the growing interest in identifying plant-derived compounds capable of regulating key molecular pathways involved in tumor survival. Understanding whether these extracts can modulate central apoptotic regulators such as Bcl-2, BAX, and Caspase-3 may provide valuable insights into their potential as sources of novel anticancer agents.

Therefore, the present study aims to investigate the pro- and anti-apoptotic effects of *O. sigmoideum* and *S. excelsa* extracts in HepG2 cells. The expression levels of Bcl-2, BAX, and Caspase-3 were evaluated using qRT-PCR to determine whether these plant extracts influence key apoptotic signaling pathways. We anticipated that the extracts would display organ-specific phytochemical compositions and corresponding differences in cytotoxicity, with *O. sigmoideum* extracts promoting apoptosis through BAX/BCL-2 dysregulation and caspase activation, while *S. excelsa* would exhibit minimal pro-apoptotic activity. By elucidating the molecular effects of these extracts on apoptosis regulation, this study aims to contribute to the understanding of their potential anticancer properties and to provide a scientific basis for future pharmacological and pharmaceutical research focused on the development of plant-derived therapeutic agents for hepatocellular carcinoma.

## 2. Results

### 2.1. Phytochemical Profiles of Extracts

The plant materials were extracted as aqueous extracts. The extraction yields, calculated on a mass-to-mass basis (% *w*/*w*), were 15.12% for the leaves of *O. Sigmoideum*, 13.81% for the bulbs of *O. sigmoideum*, and 13.48% for *S. excelsa*. GC–MS analysis revealed pronounced variations in metabolite composition among the three examined plant samples (Table 1, Table 2 and Table 3). The extract of *S. excelsa* exhibited a highly diverse chemical profile comprising 18 distinct compounds, with relative abundances ranging from 0.15% to 5.07%. The metabolite spectrum was primarily dominated by phenolic and furanone derivatives, including (pyro)-catechol (5.07%) and 3-hydroxy-4,4-dimethyldihydro-2(3H)-furanone (5.02%). Medium-chain fatty acid esters, such as pentadecanoic acid, 14-methyl-, methyl ester (3.52%), were also detected in notable amounts. In contrast, long-chain alkanes and esters were present at lower concentrations, reflecting their potential structural and energy storage roles.

The bulb extract of *O. sigmoideum* demonstrated a relatively simpler metabolite composition, containing 11 identified compounds with relative abundances between 0.32% and 3.83%. Lipid esters predominated in this fraction, notably hexadecanoic acid methyl ester (3.83%) and octadecanoic acid methyl ester (3.18%), while minor levels of phenolic and lactone derivatives were also observed. Conversely, the leaf parts of *O. Segmoideum* exhibited higher metabolic diversity and activity, encompassing 18 compounds with relative abundances ranging from 0.49% to 16.99%. The dominant metabolite in this extract was the alcohol (R,R)-butane-2,3-diol (16.99%), followed by hexadecanoic acid methyl ester (8.47%) and octadecanoic acid methyl ester (6.01%). Additional minor constituents included furanone derivatives, phenolic compounds, short-chain alkanes, and heterocyclic metabolites.

Comparative assessment across samples indicated that both hexadecanoic and octadecanoic acid methyl esters were consistently detected in all extracts, signifying a shared lipidic metabolic baseline. However, organ-specific dominance patterns were evident. The *O. sigmoideum* bulb extract was markedly lipid-enriched, consistent with its function as a storage organ. The leaf tissues exhibited a mixed composition of alcohols and fatty acids, reflecting elevated primary metabolic activity. In contrast, the *S. excelsa* extract was enriched with phenolic and furanone constituents, suggesting potential antioxidant and bioactive properties.

### 2.2. Cytotoxicity Effects

**Figure 1:**
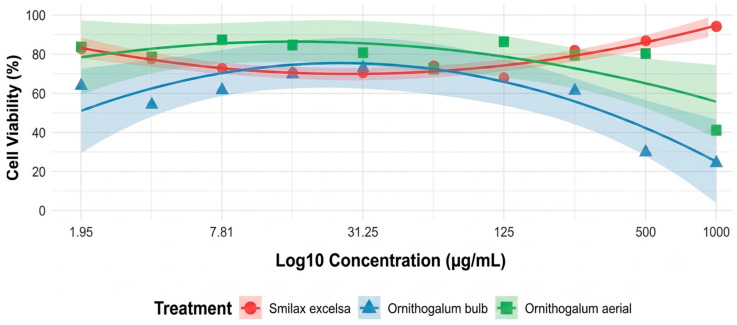
Dose-dependent effect of *Smilax excelsa* and *Ornithogalum sigmoideum* extracts on the viability of HepG2 cells.

The cytotoxic effects of *O. sigmoideum* bulb, *O. sigmoideum* leaves, and *S. excelsa* were assessed across a range of concentrations using MTT assays. A pronounced dose-dependent cytotoxic response was observed for *O. sigmoideum* bulb, with cell viability sharply decreasing to 24.42% at 1000 µg/mL. Recovery in viability occurred as concentrations decreased, reaching 86.14% at 125 µg/mL. Below this level, viability plateaued between 54.27% and 73.27% across the 62.5–1.95 µg/mL range. Nonlinear regression yielded an IC_50_ of approximately 125.57 µg/mL, and the dose–response curve exhibited a steep Hill slope of −4.20, indicative of strong inhibitory effects near the IC_50_ threshold (Figure 1).

*O. sigmoideum* leaves also showed a dose-dependent response, though with less pronounced cytotoxicity. Cell viability was reduced to 41.18% at 1000 µg/mL, but increased markedly to 80.41% at 500 µg/mL and remained relatively stable across lower concentrations (72.95–87.25%). These findings indicate significant cytotoxicity only at the highest concentration, with minimal effects at or below 250 µg/mL. The calculated IC_50_ was approximately 156.43 µg/mL, supporting a moderate cytotoxic profile with a potential therapeutic window at lower doses (Figure 1).

In contrast, *S. excelsa* displayed a more modest and somewhat atypical dose–response pattern. Surprisingly, cell viability remained high (94.40%) at the top concentration of 500 µg/mL, with the most notable cytotoxic effect observed at 62.5 µg/mL (68.06% viability). A non-monotonic trend was observed at lower concentrations (31.25–0.98 µg/mL), with viability ranging from 70.53% to 82.78%. The *S. excelsa* IC50 (304.15 µg/mL), which reflects weak overall cytotoxicity, is now explicitly described as a mathematical estimate rather than a biologically robust threshold, due to the atypical biphasic dose–response profile (Figure 1).

Collectively, these results demonstrate that *O. sigmoideum* bulb exhibits the strongest and most consistent dose-dependent cytotoxic activity, followed by *O. sigmoideum* leaves, while *S. excelsa* showed only mild cytotoxicity with potential non-linear effects at lower concentrations.

### 2.3. Gene Expression Profiles

**Figure 2:**
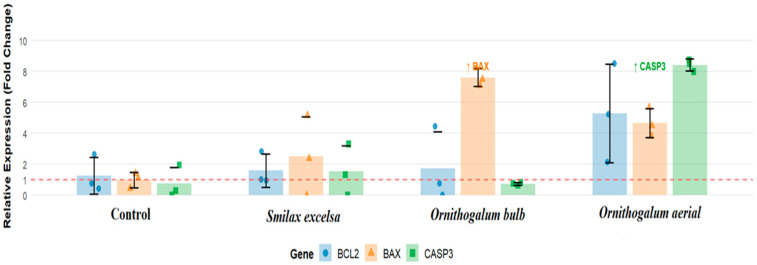
Relative mRNA expression profiles of BCL-2, BAX, and Caspase-3 in treated versus control groups.

Expression analysis of the anti-apoptotic Bcl-2 and pro-apoptotic BAX and Caspase-3 genes revealed distinct patterns among the treatment groups (Figure 2). The control group, serving as the baseline reference, demonstrated mean Ct values of 28.30, 34.99, and 29.45 for Bcl-2, BAX, and Caspase-3, respectively, and exhibited a normalized mean relative expression of 1.00, with minimal variability observed among untreated samples. While the *S. excelsa* treatment demonstrated a negligible biological effect on Bcl-2 (d = −0.13), BAX (d = 0.18), and Caspase-3 (d = 0.04) transcription, treatment with *O. sigmoideum*, both bulb and leaf parts, in contrast, displayed a distinct pattern of dysregulation.

Specifically, the bulb extract treatment significantly affects upregulation of the BCL-2 (d = 0.82) and BAX (d = 7.97) transcription, while it has no significant effect on caspase-3 expression (d = −0.39). In contrast, treatment with the *O. sigmoideum* leaf part extract has a considerable effect on all BCL-2 (d = 1.02), BAX (d = 2.91), and Caspase-3 (d = 1.44) gene transcriptomic upregulation.

**Figure 3:**
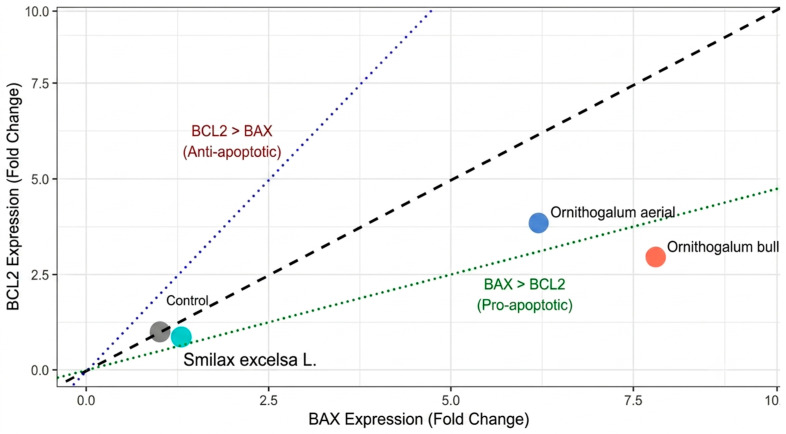
Relative BAX:BCL-2 ratio mRNA expression profiles of treated versus control groups.

To further evaluate the apoptotic balance, the BAX: Bcl-2 expression ratio was calculated for each treatment group (Figure 3). The control group exhibited a balanced ratio of 1.00, confirming baseline apoptotic homeostasis. Treatment with *S. excelsa* extract shifted the balance toward pro-apoptotic signaling with a BAX: Bcl-2 ratio of 1.51. Notably, *O. sigmoideum* bulb extract induced the most pronounced pro-apoptotic shift, with a ratio of 2.64, indicating strong activation of the mitochondrial apoptotic pathway. The *O. sigmoideum* leaf extract also promoted a pro-apoptotic state with a ratio of 1.61. These findings demonstrate that all three plant extracts alter the apoptotic rheostat in favor of cell death, with *O. sigmoideum* bulb extract exhibiting the most potent pro-apoptotic effect.

**Figure 4:**
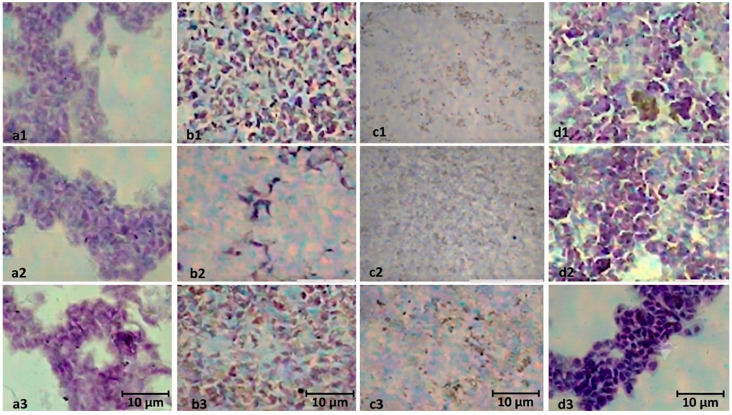
Immunocytochemistry analysis of BCL-2, BAX, and Caspase-3 protein expression. Lanes: (**a1**–**a3**) untreated control; (**b1**–**b3**) treated with *Ornithogalum sigmoideum* bulb extract; (**c1**–**c3**) treated with *Ornithogalum sigmoideum* leaf part extract; (**d1**–**d3**) treated with *S. excelsa* extract.

Immunocytochemistry analysis of BCL-2, BAX, and Caspase-3 proteins reveals a distinct pattern of expression (Figure 4). The control group showed no detectable protein expression of key apoptotic markers—Bcl-2, Bax, and Caspase-3. In contrast, treatment with *O. sigmoideum* bulb extract resulted in positive expression for Bax and Caspase-3, while Bcl-2 remained undetectable, suggesting a pro-apoptotic shift. Cells treated with *O. sigmoideum* leaf extract exhibited positive expression for all three proteins—Bcl-2, Bax, and Caspase-3—implying a complex apoptotic response with both pro-survival and pro-death signals. Interestingly, *S. excelsa* extract induced positive expression for Bcl-2 and Bax but not for Caspase-3, indicating initiation of apoptotic signaling without progression to the execution phase.

## 3. Discussion

In the present study, we characterized the phytochemical composition of *O. sigmoideum* and *S. excelsa* extracts and evaluated their dose-dependent cytotoxic effects on HepG2 cells. Importantly, this work provides the first comprehensive investigation linking the cytotoxic potential of *O. sigmoideum* and *S. excelsa* extracts with their transcriptomic influence on apoptotic enzymes, BCL-2, BAX, and Caspase-3, thereby expanding current knowledge of their phytotherapeutic potential at the molecular level.

GC–MS analysis revealed distinct phytochemical signatures among the three plant extracts, which were mirrored in their biological activities. The *O. sigmoideum* bulb and leaf extracts exhibited marked cytotoxicity in a dose-dependent manner, consistent with the known membrane-disruptive and oxidative stress–inducing properties of long-chain fatty acid esters [19,20]. In contrast, the *S. excelsa* extract, characterized by an abundance of phenolic and furanone derivatives, including (pyro)-catechol and 3-hydroxyfuranone, displayed minimal cytotoxicity [21]. These secondary metabolites are well-documented for their potent antioxidant, anti-inflammatory, and antimicrobial effects [22,23,24]. Therefore, the minimum cytotoxicity and subsequently negligible effect on apoptotic-related transcriptions can be explained, likely due to its antioxidant and cytoprotective effects.

In contrast, the bulb extract of *O. sigmoideum* presented a comparatively simpler composition dominated by lipid-derived methyl esters. The increased BCL-2 and BAX expression, accompanied by a negligible effect of caspase-3 activity, indicates that apoptosis was initiated but subsequently inhibited before the execution phase. This suggests a block at the mitochondrial level, where elevated BCL-2 counteracts BAX-mediated cytochrome c release, preventing downstream caspase activation. Such inhibition may be associated with the bioactive composition of the *O. sigmoideum* bulb extract. The extract’s dominant lipid-derived methyl esters, particularly hexadecanoic acid methyl ester, are known for their hepatoprotective and anti-inflammatory effects [25]. These compounds can stabilize cellular membranes, suppress oxidative stress, and downregulate pro-apoptotic signaling, thereby contributing to reduced caspase activation. Additionally, the presence of 1,2-benzenedicarboxylic acid diethyl ester and octadecanoic acid methyl ester, which possess anti-inflammatory and cytoprotective properties [26,27], further supports the interpretation that the extract may promote cell survival rather than apoptosis under stress conditions.

The concurrent upregulation of BCL-2, BAX, and Caspase expression in the *O. sigmoideum* leaf treatment group suggests that the apoptotic cascade was successfully activated, despite the presence of anti-apoptotic defenses. This pattern indicates that while BCL-2 expression increased as a compensatory response to cellular stress, the pro-apoptotic signaling dominated, leading to activation of the mitochondrial apoptotic pathway, potentially triggered by lipid-induced ROS generation and mitochondrial membrane destabilization [28,29,30] which results in effective initiation and execution of apoptosis.

Immunocytochemical analysis revealed protein expression patterns that partially diverged from mRNA profiles. *O. sigmoideum* bulb extract induced BAX and Caspase-3 protein expression without detectable BCL-2, suggesting post-transcriptional regulation that favors a pro-apoptotic outcome despite elevated BCL-2 mRNA. *O. sigmoideum* leaf extract yielded positive staining for all three proteins, suggesting potential coordinated activation of both mitochondrial and executioner apoptotic pathways. In contrast, *S. excelsa* induced BCL-2 and BAX but not Caspase-3, indicating potential apoptotic initiation without progression to execution.

### Limitations

Although the transcriptomic analysis of BAX, BCL-2, and CASPASE-3 provided valuable evidence of gene expression patterns consistent with pro-apoptotic signaling following treatment with *O. sigmoideum* and *S. excelsa* extracts, several limitations should be acknowledged. First, the experiments were performed solely on the HepG2 cell line, which may not fully represent the heterogeneity of tumor biology or the responses of other cancer types. Second, while GC–MS profiling identified key metabolite classes, the specific bioactive compounds responsible for the observed gene expression changes were not isolated or quantified, limiting mechanistic interpretation. Gradual column performance degradation from repeated injection of complex underivatized extracts cannot be excluded. Therefore, LC-MS/MS with reverse-phase column chemistry is recommended for comprehensive profiling. Moreover, the gene expression analysis was confined to mRNA-level observations; confirmation of apoptosis through protein expression assays would strengthen the conclusions regarding BAX/BCL-2–mediated mitochondrial signaling and caspase activation. Fourth, while our data strongly support pro-apoptosis signaling, we did not directly investigate upstream initiating events such as reactive oxygen species (ROS) generation or DNA damage. Finally, the use of relative expression fold change data for BAX, BCL-2, and Caspase3 expression provides only qualitative insight and should be complemented by qRT-PCR and enzyme activity analyses.

## 4. Materials and Methods

### 4.1. Preparation of Plant Extracts

Edible leaves of *S. excelsa* and *O. sigmoideum*, both leaves and bulb, which local people consume for their nutrients, were collected during their seasonal availability (May) from local markets in the Ordu province of Türkiye. Plant identity was confirmed by a botanist from the Department of Pharmaceutical Botany, Faculty of Pharmacy, Ankara University, through morphological examination and taxonomic verification. Voucher specimens were deposited in the university herbarium for future reference.

Following collection, plant materials were carefully washed, separated from roots and inedible fractions, and shade-dried at ambient temperature (22–25 °C) for 5–7 days to avoid thermal damage to heat-sensitive phytochemicals. The dried samples were ground into a fine powder using a laboratory mill and stored in airtight, light-protected containers at room temperature until extraction.

### 4.2. Methanolic Extraction

For each plant, 30 g of powdered material was extracted in 300 mL of absolute methanol by dynamic maceration on an orbital shaker (120 rpm, 8 h). The process was carried out in triplicate, and the resulting mixtures were filtered through Whatman No. 1 filter paper after each cycle. The combined filtrates were concentrated at 40 °C under reduced pressure using a rotary evaporator (Heidolph Laboratory, Schwabach, Germany), yielding semi-solid methanolic extracts.

The extracts were stored at –20 °C in sealed amber vials until subjected to phytochemical profiling and bioactivity testing. Extraction efficiency was calculated relative to the initial dry weight according to the following formula:

Extraction Yield (%) = (Weight of dried extract/Weight of dried plant powder) × 100

All extractions were conducted in triplicate, and results are reported as mean ± standard deviation.

Stock solutions (50 mg/mL) were prepared in distilled water containing ≤0.1% (*v*/*v*) dimethyl sulfoxide for subsequent experiments.

### 4.3. Phytochemical Analysis of Extracts

The phytochemical composition of the extracts was characterized using GC–MS to identify volatile bioactive compounds potentially responsible for their reductive properties. Analyses were performed on an Agilent 6890 gas chromatography system coupled to a mass selective detector (Agilent Technologies, Santa Clara, CA, USA). The system was equipped with an HP-5MS capillary column (30 m × 0.25 mm internal diameter, 0.25 µm film thickness; 5% phenyl methyl siloxane).

High-purity helium (99.999%) was used as the carrier gas under constant flow conditions. For GC-MS analysis, aliquots of the dried methanolic extracts were re-dissolved in HPLC-grade methanol at an appropriate working concentration and filtered through a 0.22 µm PTFE syringe filter before injection. A 1.0 µL sample was injected in splitless mode, and the injector temperature was maintained at 280 °C. The inlet pressure was set to 7.08 psi. The splitless purge was activated after a 2.0 min purge delay with a purge flow of 9.5 mL min^−1^. The gas-saver function was activated at 20 mL min^−1^ after 2.0 min.

The oven temperature program was initiated at 50 °C and held for 2 min, followed by a ramp of 5 °C min^−1^ to 240 °C with a 2 min hold, and a second ramp of 20 °C min^−1^ to 300 °C with a final hold of 2 min, resulting in a total analysis time of 47 min.

Mass spectrometric detection was carried out in full-scan mode with a solvent delay of 3.0 min. Mass spectra were acquired over an *m*/*z* range of 25–500. Ionization was performed using electron ionization (EI) at 70 eV. The ion source temperature was maintained at 230 °C, while the quadrupole temperature was set at 150 °C.

This approach captures predominantly volatile and semi-volatile constituents. Compound identification was achieved by comparing the obtained mass spectra with those available in the NIST23 mass spectral library. A similarity index (SI) threshold of ≥80%, which is generally considered indicative of reliable spectral matching, was applied to ensure reliable identification. Although retention indices (RI) were not determined due to the absence of n-alkane standards analyzed under identical chromatographic conditions, identification confidence was supported by spectral matching and comparison with previously reported GC–MS data for related species in the literature.

Relative abundances of the identified compounds were calculated using peak-area normalization without applying correction factors. All samples were analyzed in triplicate to ensure analytical reproducibility. The primary objective of the GC–MS analysis was to characterize the volatile and semi-volatile constituents of the extracts rather than to achieve comprehensive metabolomic profiling.

### 4.4. Cell Lines and Culture Conditions

For the experimental work, the human HCC cell line HepG2 (ATCC^®^ HB-8065™, Manassas, VA, USA) was selected, as it is widely recognized as a representative model for studying liver cancer research.

Cells were propagated in Dulbecco’s Modified Eagle Medium (DMEM, high glucose formulation; Gibco, Thermo Fisher Scientific, Waltham, MA, USA) enriched with 10% fetal bovine serum (FBS; Gibco, Thermo Fisher Scientific, Waltham, MA, USA) and 1% penicillin-streptomycin solution (10,000 U/mL penicillin, 10 mg/mL streptomycin; Gibco). Cultures were maintained in T-75 cm^2^ flasks (Corning, NY, USA) under controlled environmental conditions—37 °C, 5% CO_2_, and 95% humidity—to closely mimic the physiological microenvironment. Experiments were performed using cells between passages 10–15. Cells were maintained under standard aseptic conditions, and no morphological or growth abnormalities indicative of contamination were observed during the study period.

### 4.5. Cell Maintenance and Subculturing

Cells were observed daily under an inverted phase-contrast microscope (Olympus CKX53, Tokyo, Japan) to assess morphology and confluency. Healthy HepG2 cells were characterized by their typical epithelial-like morphology with clear cell boundaries and uniform adherence. Confluency was estimated visually, and cells were subcultured when they reached approximately 70–80% confluency every 2–3 days to prevent overgrowth and maintain physiological behavior.

The passaging process began with the removal of spent medium, followed by rinsing the adherent monolayer with phosphate-buffered saline (PBS; pH 7.4). Cells were then enzymatically detached using 0.25% trypsin–EDTA solution (Gibco). The culture flasks were incubated at 37 °C for 2–3 min with gentle agitation every minute to ensure even detachment. Trypsinization was monitored microscopically, and the reaction was immediately terminated upon complete cell rounding and detachment by adding an equal volume of complete culture medium containing 10% FBS to neutralize enzymatic activity. Prolonged trypsin exposure (>5 min) was strictly avoided to prevent cell membrane damage and maintain optimal viability. The cell suspension was centrifuged at 200 × g for 5 min, after which the pellet was resuspended in fresh medium and reseeded at the desired density. Following trypsinization, cell viability was immediately assessed using trypan blue exclusion, and only cultures with viability >90% were used for subsequent experiments. To maintain experimental consistency and limit variability, only cells within passages 5–20 were used in downstream assays.

### 4.6. Cell Viability and Counting

The trypan blue exclusion method was employed to evaluate both cell density and viability. Briefly, equal volumes of cell suspension and 0.4% trypan blue solution (Gibco) were mixed, and the proportion of viable (unstained) to non-viable (blue-stained) cells was determined using a Thoma hemocytometer under light microscopy, and counted within 2–3 min.

Only cultures displaying viability levels above 90% were considered suitable for subsequent experiments.

### 4.7. Cytotoxicity Assay and Half-Maximal Inhibitory Concentration Determination

The cytotoxic potential of *S. excelsa* leaf extracts and both leaf and bulb extracts of *O. sigmoideum* was assessed against HepG2 cells using the 3-(4,5-dimethylthiazol-2-yl)-2,5-diphenyltetrazolium bromide (MTT) assay [31]. This method relies on the ability of viable cells to reduce the yellow tetrazolium salt [3-(4,5-dimethyl-2-thiazol-2-yl)-2,5-diphenyl-2H-tetrazolium bromide] into insoluble purple formazan crystals via mitochondrial dehydrogenase activity, providing a quantitative measure of cell viability.

HepG2 cells were seeded at 1.5 × 10^4^ cells per well into 96-well plates in 50 µL of medium and allowed to adhere for 24 h at 37 °C. The medium was then replaced with 150 µL of fresh complete medium prior to treatment. For plate setup, the first column contained medium alone (blank control), while the second column served as a cell control (cells with culture medium only). The remaining wells were treated with serial dilutions of each plant extract, ranging from 1000 to 1.95 µg/mL.

Cells were exposed to the extracts for 48 h under standard culture conditions (37 °C, 5% CO_2_, 95% humidity). Following treatment, 20 µL of MTT solution (5 mg/mL in PBS) was added to each well and incubated for 4 h to allow formazan crystal formation. To solubilize the crystals, 50 µL of 10% (*w*/*v*) sodium dodecyl sulfate (SDS) was added, and plates were kept overnight at room temperature in the dark.

Given that the first column contained a blank control, to minimize the border effect, plates were sealed with Parafilm during incubation to minimize evaporation, the microplate reader was pre-warmed before measurement.

Absorbance was measured at 570 nm using a microplate reader, with background correction applied using blank wells. Percent viability was calculated relative to untreated cell controls. Each experiment was conducted in six replicates to ensure statistical robustness.

Dose–response curves were generated by plotting cell viability against the logarithm of extract concentrations. The half-maximal inhibitory concentration (IC_50_) for each extract was derived from the linear region of the curve by regression analysis.

### 4.8. Gene Expression Analysis

#### 4.8.1. RNA Isolation and cDNA Preparation

Total RNA was extracted from HepG2 cells treated with selected plant extracts using the Hybrozol reagent (Hibrigen Biotechnology, Kocaeli, Türkiye), which employs a guanidinium thiocyanate–phenol–chloroform mechanism comparable to TRIzol. Cells were seeded at 1 × 10^6^ cells per well in 6-well plates and incubated for 48 h under standard conditions. Untreated cells were included as negative controls, and all experiments were performed in triplicate for reproducibility.

Following incubation, the medium was discarded and Hybrozol reagent was applied (1 mL per 10 cm^2^ culture area). After a 5 min lysis at room temperature, cell lysates were transferred into RNase-free microcentrifuge tubes. Chloroform (0.2 mL per 1 mL Hybrozol) was added, vortexed for 15 s, and samples were allowed to stand for 2–3 min before centrifugation (12,000× *g*, 8 min, 4 °C). The aqueous phase was carefully recovered, and RNA was precipitated with isopropanol (500 µL). After 10 min incubation at room temperature, samples were centrifuged again (12,000× *g*, 8 min, 4 °C). The RNA pellet was washed three times with 75% ethanol, air-dried, and dissolved in 40 µL of DEPC-treated water. Extracted RNA was stored at −80 °C until further use.

All consumables and equipment were pre-treated with DEPC water and autoclaved to maintain an RNase-free environment.

#### 4.8.2. RNA Quality Control

Concentration and purity were measured using a NanoDrop™ spectrophotometer (Thermo Fisher Scientific, Waltham, MA, USA). Only samples with an A260/280 ratio of 1.8–2.0 and an A260/230 ratio above 2.0 were accepted. Integrity was confirmed by 1% agarose gel electrophoresis, where intact RNA exhibited distinct 28S and 18S rRNA bands without visible degradation.

#### 4.8.3. cDNA Synthesis

Messenger RNA was reverse-transcribed into complementary DNA (cDNA) using the iScript™ cDNA Synthesis Kit (Bio-Rad, CA, USA) according to the manufacturer’s instructions. cDNA samples were either used immediately for qRT-PCR analysis or stored at −20 °C until needed. All steps were performed with RNase-free reagents and on ice to preserve RNA integrity.

### 4.9. Qualitative mRNA Expression Analysis

To investigate transcriptional changes associated with apoptosis, the relative expression levels of BAX, BCL-2, and Caspase-3 were examined using qRT-PCR in treated and untreated cell populations.

The primer sequences for each target gene are provided in Table 4 retrived from previous studies [32,33]. These genes were chosen for their central involvement in apoptotic signaling, allowing assessment of whether the treatments modulated pro- or anti-apoptotic pathways. To normalize expression values and correct for variability across samples, β-actin and GAPDH were employed as internal controls. Both housekeeping genes were selected based on their stable expression under the experimental conditions, ensuring reliable relative quantification.

qRT-PCR was performed on an Applied Biosystems QuantStudio 5 Real-Time PCR system using the RT^2^ SYBR^®^ Green qPCR Mastermix Low ROX (Qiagen, Germantown, MD, USA). Reaction setup adhered to the manufacturer’s specifications for final volume and reagent concentrations. For each well, 120 ng of cDNA was used as a standardized template to maintain consistency among samples.

Thermal cycling was carried out in three phases, as outlined in Table 5. Every sample was analyzed in triplicate, ensuring data reliability and reducing the likelihood of pipetting-related variability.

#### 4.9.1. Relative Quantification and Data Analysis

Gene expression was quantified using the 2^−ΔΔCt^ method, enabling comparison between treated and untreated samples after normalization to the selected housekeeping genes. For each target gene, the cycle threshold (Ct) values were first normalized to the geometric mean of β-actin and GAPDH (ΔCt). The ΔΔCt values were then calculated by comparing treated samples to their corresponding controls. Relative fold changes were derived from these values and visualized to determine whether treatment induced upregulation or downregulation of the genes of interest.

#### 4.9.2. Protein Expression Analysis

Protein expression of BAX, BCL-2, and Caspase-3 was assessed at the cellular level using immunocytochemical staining in treated cell groups. After the experimental treatments, cells were rinsed twice with phosphate-buffered saline (PBS) and fixed in 4% paraformaldehyde for 15 min at room temperature. Cell membranes were then permeabilized with 0.1% Triton X-100 prepared in PBS for 10 min. To reduce nonspecific antibody interactions, a blocking step was performed using 5% bovine serum albumin (BSA) in PBS for 1 h.

Subsequently, cells were exposed to primary antibodies specific for BAX, BCL-2, and Caspase-3 diluted in the blocking solution, and incubated overnight at 4 °C. Following PBS washes, HRP-linked secondary antibodies were applied for 1 h at room temperature. Antigen–antibody complexes were detected using 3,3′-diaminobenzidine (DAB) as the chromogen, and nuclei were counterstained with hematoxylin.

Finally, coverslips were mounted with an aqueous-based mounting medium and visualized under a light microscope. The presence of immunoreactivity was assessed qualitatively by comparing staining patterns with control samples and was recorded as positive (+) or negative (−) based on observable cytoplasmic or nuclear staining.

### 4.10. Data Analysis

The relative abundance of each identified compound was expressed as a percentage of the total chromatographic peak area (Area%). The IC_50_ was determined from dose–response curves using nonlinear regression analysis. The descriptive statistics for the relative expression levels of three apoptosis-related genes (Bcl-2, BAX, and Caspase-3) were analyzed across experimental groups (control, *S. excelsa*, *O. sigmoideum* bulb, and *O. sigmoideum* leaves), with each group consisting of three biological replicates (*N* = 3 per group; Total *N* = 12). The data are presented as means, standard deviations (SD), standard errors (SE), 95% confidence intervals (CI), and minimum/maximum values. The effect of each treatment upon target genes’ relative gene expression fold change compared to the control group was evaluated by Cohen’s D.

## 5. Conclusions

The study demonstrates that extracts from *O. sigmoideum* (both bulb and leaf parts) and *S. excelsa* have distinct phytochemical profiles, which directly correlate with their differential cytotoxic and pro-apoptotic effects on HepG2 liver cancer cells. While *S. excelsa* shows minimal cytotoxicity and no significant effect on apoptosis, likely due to its antioxidant-rich composition, with further validation through multi-line and in vivo studies, *O. sigmoideum*-derived compounds, particularly leaves, exhibit significant cytotoxicity and a transcriptomic profile that is consistent with apoptotic pathway activation in HepG2 cells, positioning it as a candidate for further mechanistic investigation.

While these preliminary data support the potential pro-apoptotic properties of *O. sigmoideum*, they should be interpreted as a foundation for future mechanistic studies. Future research should incorporate multiple cancer cell lines and in vivo tumor models, coupled with advanced metabolomic characterization, to validate these preliminary findings. Additionally, evaluating potential synergistic or protective interactions between these plant extracts and standard chemotherapeutic agents would further clarify their therapeutic relevance and safety profiles. Also, a non-tumorigenic hepatic cell line to calculate the selectivity index (SI) in future experiments should be strongly considered.

## Figures and Tables

**Table 1:** Phytochemical contents of *Smilax excelsa*.

Library ID	Retention Time (Minute)	Area Percentage	CAS	Similarity Index %
(pyro)-catechol	14.5494	5.0672	000120-80-9	91
3-Hydroxy-4,4-dimethyldihydro-2(3H)-furanone	9.7942	5.0242	000599-04-2	86
Pentadecanoic acid, 14-methyl-, methyl ester	31.66	3.524	005129-60-2	99
Octadecanoic acid, methyl ester	35.4305	2.5312	000112-61-8	99
Phenol	8.3111	1.1019	000108-95-2	94
Hexadecane	24.7349	0.6191	000544-76-3	98
Tetradecane	19.8903	0.6083	000629-59-4	94
(pyro)-catechol	14.7114	0.1495	000120-80-9	91

All compounds tentatively identified by NIST23 spectral matching (SI ≥ 80%) only; no experimental retention indices determined. Independent verification required.

**Table 2:** Phytochemical contents of *Ornithogalum sigmoideum* bulb.

Library ID	Retention Time (Minute)	Area Percentage	CAS	Similarity Index (%)
Hexadecanoic acid, methyl ester	31.6595	3.8324	000112-39-0	99
Octadecanoic acid, methyl ester	35.4312	3.1827	000112-61-8	99
Propanoic acid, 2-methyl-, 3-hydroxy-2,4,4-trimethylpentyl ester	19.241	0.8616	074367-34-3	83
Tetradecane	19.8876	0.8594	000629-59-4	96
Hexadecane	24.7309	0.8566	000544-76-3	96
.alpha.-Methyl-.gamma.-crotonolactone	6.7488	0.8033	022122-36-7	91
2,3-Dihydro-3,5-dihydroxy-6-methyl-4H-pyran-4-one	12.8506	0.4775	028564-83-2	87
Octadecane	29.1032	0.3672	000593-45-3	96
Pentacosane	22.2911	0.3211	000629-99-2	83

All compounds tentatively identified by NIST23 spectral matching (SI ≥ 80%) only; no experimental retention indices determined. Independent verification required.

**Table 3:** Phytochemical contents of the leaves of *Ornithogalum sigmoideum*.

Library ID	Retention Time (Minute)	Area Percentage	CAS	Similarity Index %
(R,R)-Butane-2,3-diol	4.212	16.9992	000513-85-9	91
Hexadecanoic acid, methyl ester	31.6589	8.4725	000112-39-0	99
Octadecanoic acid, methyl ester	35.4313	6.0106	000112-61-8	99
Tetradecane	19.8866	2.1104	000629-59-4	97
Hexadecane	24.7344	1.6385	000544-76-3	96
2(3H)-Furanone, dihydro-	6.6303	1.1757	000096-48-0	91
Octadecane	29.1068	1.1282	000593-45-3	96
Acetamide	3.9099	0.8353	000060-35-5	80
2-Pyrrolidone	10.6674	0.8351	000616-45-5	83
Phenol	8.3734	0.7512	000108-95-2	94
Pyrazine, tetramethyl-	11.3005	0.5138	001124-11-4	90
Phenol, 2-methoxy-	11.3866	0.491	000090-05-1	93

All compounds tentatively identified by NIST23 spectral matching (SI ≥ 80%) only; no experimental retention indices determined. Independent verification required.

**Table 4:** The primer sequences of BAX, BCL-2, and Caspase-3, and two housekeeping genes—β-actin and GAPDH.

Gene ID		Sequence (5′ → 3′)	GenBank Access Number
BCL-2	F	GGTGGGGTCATGTGTGTGG	NM_000657
	R	CGGTTCAGGTACTCAGTCATCC	
BAX	F	CCCGAGAGGTCTTTTTCCGAG	NM_138763
	R	CCAGCCCATGATGGTTCTGAT	
Caspase3	F	CATGGAAGCGAATCAATGGACT	NM_004346
	R	CTGTACCAGACCGAGATGTCA	
β-Actin	F	GAGCGCGGCTACAGCTT	NM_001101
	R	TCCTTAATGTCACGCACGATTT	
GAPDH	F	GGAGCGAGATCCCTCCAAAAT	NM_001256799
	R	GGCTGTTGTCATACTTCTCATGG	

**Table 5:** The thermal cycling protocol.

Stage	Step	Temperature	Duration	Cycles
1. Initial Enzyme Activation	-	95 °C	3 min	1 cycle
2. Amplification Cycles	Denaturation	95 °C	15 s	40 cycles
2. Amplification Cycles	Annealing & Extension	60 °C	1 min	40 cycles
3. Melting Curve Analysis	Step 1	95 °C	15 s	1 cycle
3. Melting Curve Analysis	Step 2	60 °C	1 min	
3. Melting Curve Analysis	Step 3	95 °C	15 s	

## Data Availability

Data is available upon request from author.
